# Pancreatic Anastomosis Disruption Seven Years Postpancreaticoduodenectomy

**DOI:** 10.1155/2010/436739

**Published:** 2010-08-16

**Authors:** Walid Faraj, Zaki Abou Zahr, Deborah Mukherji, Ahmad Zaghal, Mohamed Khalife

**Affiliations:** HPB and Liver Transplant Unit, Department of Surgery, American University of Beirut Medical Center, 961 Beirut, Lebanon

## Abstract

We are reporting a case of a 22 year-old female patient, who underwent a pancreaticoduodenectomy previously for a solid-pseudopapillary neoplasm of the pancreas and was re-admitted seven years later with a pancreatic leak following disruption of the pancreatico-jejunal anastomosis. Exploratory laparotomy revealed a large collection at the level of the pancreatic anastomosis with major disruption of the pancreatico-jejunal anastomosis. The pancreatic stump was refreshed as well as the jejunal site and a duct to mucosa anastomosis was performed. She remains well with a follow up of 18 months.

## 1. Case Report

A 22-year-old female patient, who underwent a pancreaticoduodenectomy seven years ago for a solid pseudopapillary neoplasm of the pancreas, was admitted with a pancreatic leak following disruption of the pancreatico-jejunal anastomosis. 

At age 15, she presented with symptoms of postprandial epigastric pain and heaviness; physical examination was unremarkable. Family history was positive for pancreatic malignancy (maternal grandfather and grandmother). Ultrasound and computed tomography (CT) imaging of the abdomen revealed a 3.8 × 3 cm mass in the head of the pancreas with no involvement of mesenteric vessels. Endoscopic ultrasound confirmed the presence of the mass. CT-guided fine needle aspiration revealed a solid papillary cystic epithelial neoplasm. 

A pylorus-preserving pancreaticoduodenectomy was performed with the following reconstruction: the pancreatic anastomotic reconstruction was via a loop of jejunum which was anastomosed to the pancreas in a telescoping fashion using 3/0 silk sutures. The biliary anastomosis was performed using 4/0 Polydioxanone (PDS) sutures in an interrupted fashion end-to-side with the same jejunal loop. The gastro-jejunal anastomosis was performed in an end-to-side fashion using 3/0 PDS. The operative time was 5.5 hours with minimal blood loss. Histological examination of the resected specimen showed a solid papillary tumor with four lymph nodes negative for malignancy. The patient made an excellent recovery with no complications and was discharged home after ten days. She was followed up with abdominal CT scans every six months for the first two years followed by annual scans with no evidence of tumor recurrence.

Seven years postsurgery, she presented with a 4-day history of epigastric pain and discomfort, nausea, and vomiting. On physical examination, her abdomen was distended and dull on percussion with minimal bowel sounds. The biochemical profile included serum aspartate aminotransferase (AST) 70 IU/L, bilirubin (total) 10 umol/L, gamma glutamyl transferase 68 IU/L, alkaline phosphatase 106 IU/L, amylase 300 U/L, lipase 220 U/L, white blood count 9800, and hemoglobin 10 g/dl.

**Figure 1:**
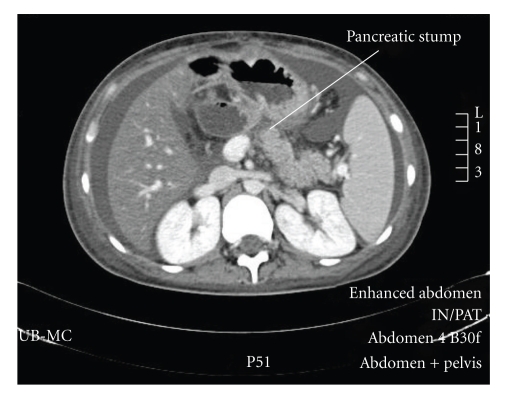
Abdominal CT scan showing the disconnected pancreatic stump with fluid in the abdomen.

Abdominal ultrasound revealed a large amount of free fluid in the abdomen, with no evidence of focal collection or recurrence of the tumor. There was no evidence of hepatic or portal venous system thrombosis. A percutaneous drain was inserted, and the intraabdominal fluid amylase was 7500 U/L and lipase 7200 U/L. The next day she started to complain of severe left-upper quadrant pain radiating to the epigastric area with bilious vomiting. CT scan of the abdomen showed a large collection at the level of the body of the pancreas with a hyperdense area suspicious for active extravasation of contrast from the bowel (Figure 1). Exploratory laparotomy revealed a large collection at the level of the pancreatic anastomosis with major disruption of the pancreaticojejunal anastomosis. The pancreatic stump was refreshed as well as the jejunal site, and a duct-to-mucosa anastomosis was performed using 4/0 PDS. The patient made an excellent recovery and was discharged home after 14 days. She remains well with a followup of 18 months.

## 2. Discussion

Pancreaticoduodenectomy is the standard procedure for resection of benign and malignant diseases of the pancreatic head. The early operative mortality rate was up to 20% [1–3]; with the advent of modern surgery and postoperative care, the mortality rate has dropped to 2%–5%, and the morbidity rate ranges from 10 to 40% [4–12].

Improvements in outcome are related to advances in surgical techniques, anesthesia, intensive care, and interventional radiology.

The most common postoperative complications are hemorrhage, wound infection, pancreatic anastomotic leak, fistula, and delayed gastric emptying. Pancreatic anastomotic leak remains the leading cause of morbidity with an incidence reaching 20% even in specialized centers [13–17]. Complicated pancreatic anastomotic leak may lead to sepsis and hemorrhage with a mortality rate of up to 40% [8, 13, 14, 18].

To our knowledge, this is the first report in the literature of a pancreatic leak 7 years postpancreaticoduodenectomy. Most pancreatic leaks have been reported as a complication occurring within 10 days postoperatively [5, 11, 19]. The presentations of late leaks have been reported as occult fistula [16] within a few months postoperatively. 

The perioperative factors which predispose to pancreatic leak include older age (more than 65 years), preoperative jaundice [4], large intraoperative blood loss [20], intraoperative blood transfusion, prolonged operative time (>8 hrs), diabetes [17], low patient volume per surgeon, and ampullary or duodenal disease [21].

Differences in surgical techniques in pancreatic anastomosis have led to controversies regarding the best technique to use. Several techniques have been described, and these include duct-to-mucosa pancreaticojejunostomy (PJ), end-to-end anastomosis with invagination of the pancreatic stump in a loop of jejunum, pancreaticogastrostomy (PG) with end-to-side and end-to-end anastomosis with invagination. Many studies showed a decreased leak rate with PG [22, 23] than with PJ. In contrast, Yeo et al. [5] showed that there is no difference in pancreatic leak rate following both PJ and PG. He reported high post-PG pancreatic leak rate reaching 12.3%. Kim et al. [9] found no significant differences in overall leak rate between PJ and PG though the majority of cases were pylorus preserving. He showed that the overall duct-to-mucosa technique has a significantly lower risk of leak as compared to invagination technique (3.2% versus 17.5%). This was seen in the PG group and not in the PJ group, where in the PJ group no significant difference was established between the two techniques.

We propose that the cause of the very late anastomotic disruption we have reported is likely to be secondary to pancreatic duct obstruction, followed by recurrent pancreatitis, ischemia, and fibrosis. In conclusion, early pancreatic leaks are fairly common and can reach up to 20% even in specialized centers whereas late pancreatic anastomotic leak is a rare phenomenon.
